# Plasmon coupled Fabry-Perot lasing enhancement in graphene/ZnO hybrid microcavity

**DOI:** 10.1038/srep09263

**Published:** 2015-03-19

**Authors:** Jitao Li, Mingming Jiang, Chunxiang Xu, Yueyue Wang, Yi Lin, Junfeng Lu, Zengliang Shi

**Affiliations:** 1 State Key Laboratory of Bioelectronics, School of Biological Science and Medical Engineering, Southeast University, Nanjing 210096, China; 2 School of Physics and Electromechanical Engineering, Zhoukou Normal University, Zhoukou 466001, China; 3 State Key Laboratory of Luminescence and Applications, Changchun Institute of Optics, Fine Mechanics and Physics, Chinese Academy of Sciences, Changchun 130033, China

## Abstract

The response of graphene surface plasmon (SP) in the ultraviolet (UV) region and the realization of short-wavelength semiconductor lasers not only are two hot research areas of great academic and practical significance, but also are two important issues lacked of good understanding. In this work, a hybrid Fabry-Perot (F-P) microcavity, comprising of monolayer graphene covered ZnO microbelt, was constructed to investigate the fundamental physics of graphene SP and the functional extension of ZnO UV lasing. Through the coupling between graphene SP modes and conventional optical microcavity modes of ZnO, improved F-P lasing performance was realized, including the lowered lasing threshold, the improved lasing quality and the remarkably enhanced lasing intensity. The underlying mechanism of the improved lasing performance was proposed based on theoretical simulation and experimental characterization. The results are helpful to design new types of optic and photoelectronic devices based on SP coupling in graphene/semiconductor hybrid structures.

In recent years, short-wavelength semiconductor lasers have drawn much attention due to their unique properties and wide applications in optoelectronic devices^1^,^2^. Among the wide band gap semiconductor materials, zinc oxide (ZnO) has been considered as a versatile building blocks for photonic applications and demonstrated as a competitive candidate to realize ultraviolet (UV) lasing based on its wide direct band gap (3.37 eV) and high exciton binding energy (60 meV)^3^,^4^. So far, UV lasing of ZnO has been observed in various micro/nanostructures through random^5^,^6^, Fabry-Perot (F-P)^7^,^8^, and whispering-gallery mode (WGM)^9^,^10^,^11^ resonant approaches. Nevertheless, it is difficult to realize high lasing output or miniaturized dimension lasers with high efficiency based on micro/nanostructures due to the big cavity losses and the diffraction limit. Recently, surface plasmon (SP) has attracted intense interests in a wide scope of scientists, ranging from physicists, chemists and material scientists to biologists, due to its particular properties and many applications, such as surface-enhanced Raman scattering (SERS)^12^,^13^, biosensing^14^, photoresponse^15^ and surface-enhanced fluorescence^16^,^17^,^18^. Though noble metals have been regarded as the best available plasmonic materials for the improved photonic applications^19^,^20^,^21^,^22^, the metal SP is inconveniently tunable in fixed devices and generally has large Ohmic losses. These unfavorable factors astrict the flexible design and development of novel functional photonic materials and devices.

Graphene, a flat monolayer of carbon atoms tightly packed into a honeycomb lattice, has attracted much attention ever since its discovery in 2004 due to its remarkable properties and potential applications^23^,^24^,^25^,^26^. Nair and Sun *et al.* have demonstrated that graphene has about 2.3% absorption in visible region but an abnormal absorption in the UV region due to its unique electronic structure and the Dirac-fermionic energy dispersion^25^,^27^. This abnormal absorption indicates the excitation of the spectrally broadened SP modes in graphene. The *π* → π* transition would response to the excitation in low-energy UV region according to the theoretical research by Eberlein and Trevisanutto *et al*^28^,^29^. Koppens *et al.* further revealed graphene-induced strong light-matter interaction and the corresponding optical field confinement^30^. So it is significant to use graphene to improve the optical performance and even develop new types of optic and photoelectronic devices. For graphene/ZnO system, a representative research has revealed a SP dispersion relation and demonstrated an important role in the response of the resonant excitation of graphene plasmon due to the abnormally increased UV absorption^31^. Similarly, Chen *et al.* reported an enhancement of the random lasing action in a nanocomposite of reduced graphene oxide nanoflakes and ZnO nanorods^32^. Though these reports have indicated that the graphene SP can be used to improve the performance of optoelectronic devices in UV region and assumed the action of graphene SP, the mechanism is unclear, even in some controversy^33^. Theoretical simulation and experimental observation are really needed to support the assumed SP effect at the interface of graphene/ZnO.

In this work, to provide sufficient evidence of graphene SP's response in UV region and extend its functionality, a hybrid F-P microcavity consisted of monolayer graphene covered ZnO microbelt was designed and fabricated. ZnO microbelt with rectangular cross-section can be treated as an intrinsic F-P type microresonators, whilst as the gain medium. Graphene covered on the surface of the microbelt acted as the external modulation. Graphene SP excited along the interface of graphene/ZnO could modulate the local field, which can compensate the optical loss and make the radiative photons be better imprisoned within the F-P microresonators. Through the coupling between graphene SP modes and conventional optical microcavity modes, obviously improved F-P lasing performance was achieved. The interaction mechanism was investigated through theoretical simulation and experimental characterization. The results are helpful not only to understand the interaction between graphene and semiconductor under the optical excitation in physics but also to inspire novel design of graphene-based optic and photoelectronic devices in technology.

## Results and Discussion

**Figure 1:**
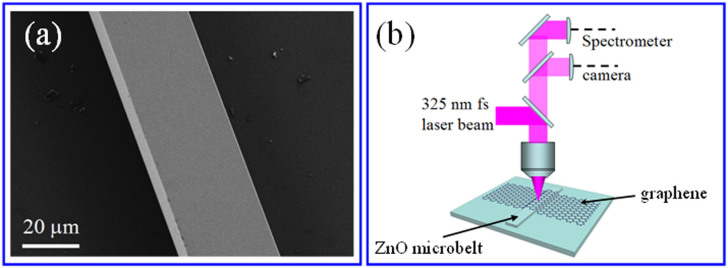
(a) SEM image of an individual ZnO microbelt. (b) The schematic diagram for the optical measurement setup.

ZnO microbelts were synthesized by a vapor phase transport (VPT) method and Figure 1(a) shows the SEM image of the ZnO microbelt used in this experiment. It can be seen that the microbelt has flat and smooth side surfaces and rectangular cross-section with width and thickness of the cross-section about 30 μm and 5 μm, respectively. The measurement setup for the optical experiment is schematically shown in Figure 1(b), where the excitation lasing was focused by a microscope objective (40×) to a spot size of about 15 μm on the samples. The PL signals were collected by the same microscope objective and recorded by a CCD detector.

**Figure 2:**
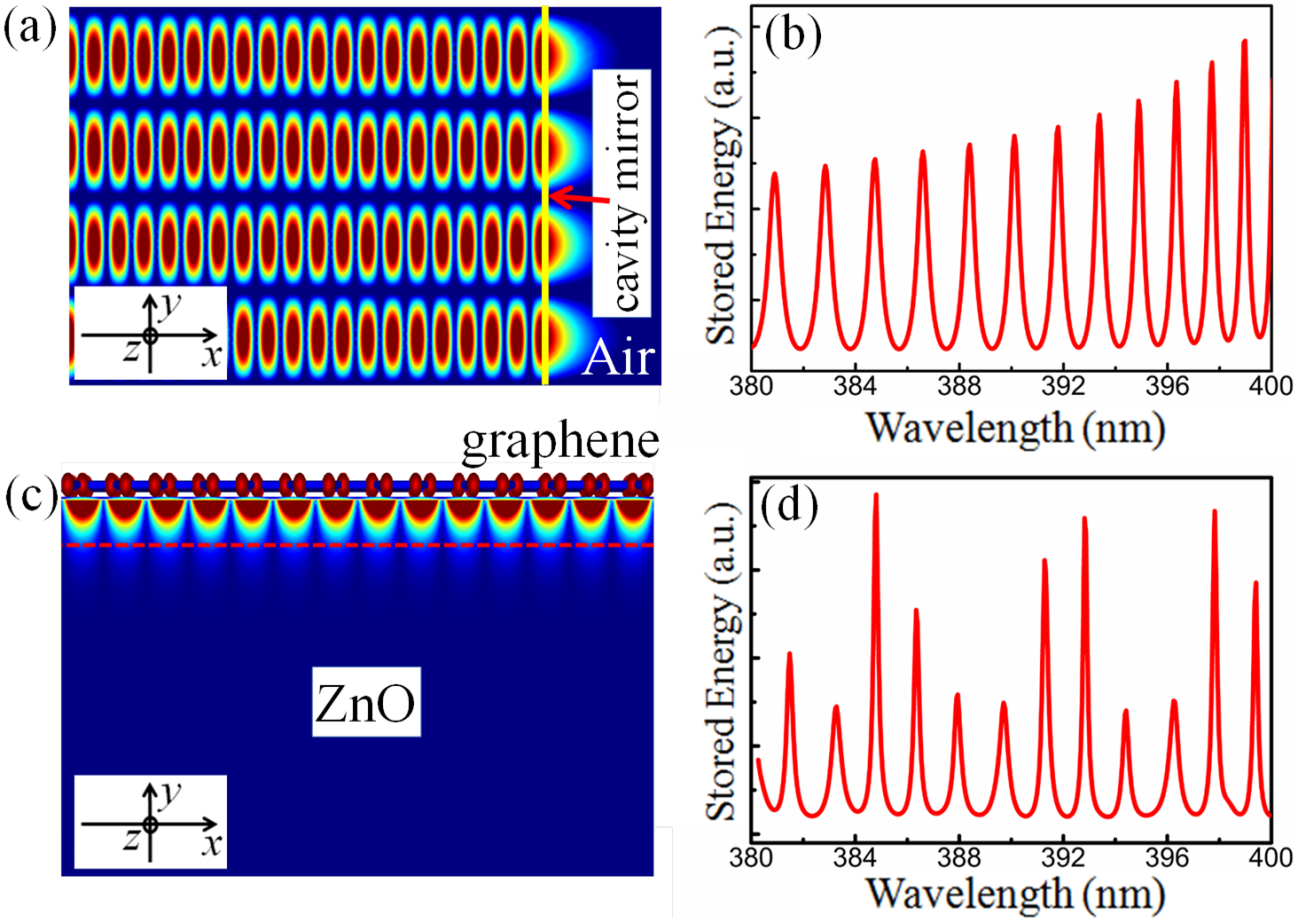
(a) The standing-wave pattern of the optical field in the *x-y* plane (the cross-section plane) of the bare microbelt. The yellow line denotes the interface of ZnO/air, i.e. the cavity mirror of the microcavity, and the length direction of the microbelt goes along the *z* axis. (b) The resonance spectrum of the stored energy for the bare microcavity with air/ZnO interface. (c) Graphene SP excited along the interface of graphene/ZnO. The region between graphene and the red dash line denotes the crossover region of graphene SP evanescent wave field and the optical microcavity modes. (d) The resonance spectrum of the stored energy for the hybrid microcavity.

By using a finite difference time domain (FDTD) method, the optical field distribution and the mode structure in the bare and the hybrid microcavity were simulated. Figure 2(a) shows the optical field distribution in the *x-y* plane (i.e. the cross-section) of the bare ZnO microcavity. In Figure 2(a), the yellow line denotes the interface of ZnO/air, which can act as mirror of the microcavity and provide feedback of the photons oscillation. From the simulation, it can be seen that the photons of the cavity modes could be trapped inside the cavity body, and the corresponding standing wave pattern of the oscillation was formed between the two lateral sides of the microcavity. Perfect standing wave field distribution was observed and the wave beam could propagate back and forth between the two lateral sides of the microcavity to form the F-P resonance mechanism. It should be noted that a proper of the light leaks out of the cavity and transfers to free space, indicating some optical loss at the ZnO/air interface. Figure 2(b) shows spectrum of the stored energy as function of the wavelength for the bare ZnO microcavity. In doing this, we use a source with a variable frequency to excite the modes in the cavity. The calculation result demonstrates that the stored energy of different modes has almost the same intensity, the small deviation between them come from the small variation in the refractivity at different mode wavelengths. The calculation result also demonstrates that the full width at half maximum (FWHM) of the resonant modes are broad, meaning the poor lasing quality of the bare F-P microcavity due to the optical loss shown in Figure 2(a). The aforementioned discussion gives an important signal that the ZnO microbelt can be used to realize F-P lasing based on the two lateral sides of the cavity acted as mirrors, but with relatively poor lasing quality due to the optical loss of the bare microcavity. This will be directly observed and discussed in the experimental observation.

As aforementioned, in order to improve the performance of conventional optical microcavities, a kind of hybrid plasmonic microcavity system was proposed to achieve the coupling of SP modes with conventional optical cavity modes^34^,^35^. Likewise, graphene SP is also expected to strengthen the light-matter interaction due to SP provided subwavelength confinement, guided modes and so on^36^,^37^,^38^. As shown in Figure 2(c), after graphene was covered on the ZnO microbelt, its abnormal UV absorption can induce collective oscillation of the two-dimensional electron gas, which can promote multimode oscillation and lead to localized SP excited along the interface of graphene/ZnO, and a crossover region will exist along the interface. The excited evanescent wave field would be confined in the crossover region and strongly localized at subwavelength scale. Therefore, the crossover region can provide a platform to achieve the coupling between the SP modes and conventional F-P resonant modes, which would modulate the total energy distribution and the oscillation process in the microcavity. Figure 2(d) illustrates the resonance spectrum of the stored energy for the hybrid F-P microcatity. It demonstrates that the FWHM of each resonant mode become narrower, reflecting the improved lasing quality of the hybrid microcavity relative to that of the bare one. This indicates the coupling between graphene SP and ZnO emission, and supports the experimental observation on the PL enhancement discussed later.

**Figure 3:**
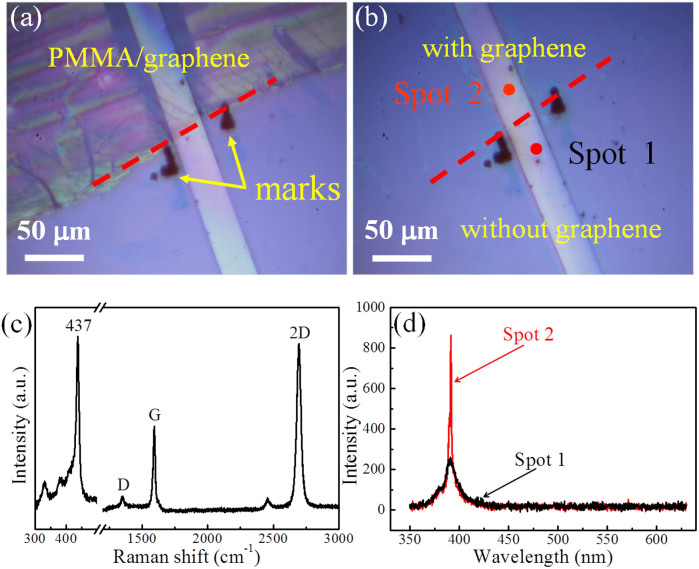
(a, b) Optical images of the ZnO microbelt partially covered with monolayer graphene: (a) before and (b) after dissolving of PMMA. The red dash line denotes the boundary of the areas with and without graphene. (c) Raman spectrum from spot 2 of the graphene/ZnO microbelt hybrid microcavity. (d) PL spectra from the two spots on the microbelt with (spot 2) and without (spot 1) graphene under the same excitation condition.

Experimentally, in order to investigate the influence of graphene on the optical behaviors of the ZnO microbelt, a hybrid microstructure was fabricated by partially covering ZnO microbelt with monolayer graphene. Figure 3(a) shows optical image of the fabrication. For comparison, two marks were ablated on the substrate near the two lateral sides of the microbelt at the boundary of the PMMA/monolayer graphene. By this way, one can easily find the part of the ZnO microbelt with/without graphene, which contacts tightly on top surface of the microbelt after dissolving of PMMA, as shown in Figure 3(b).

To assess the coverage state of graphene on the ZnO microbelt, Raman spectrum from the graphene covered ZnO microbelt was measured, as shown in Figure 3(c). The spectrum shows not only the typical E_2_(high) modes of wurtzite ZnO at 437 cm^−1^, but also the sharp and intensive 2D peak and G peak of graphene. The strong E_2_ mode demonstrates the good crystal quality of the ZnO microbelt. The sharp 2D peak and G peak of graphene present single-Lorentzian curves with FWHM of 31 cm^−1^ and 14 cm^−1^, respectively, and the intensity ratio of 2D mode to G mode (I_2D_/I_G_) is ~2.2, indicating the high quality of the monolayer graphene. Based on these characteristics, we can believe that the monolayer graphene was successfully covered on the ZnO microrbelt and the graphene/ZnO hybrid microcavity was fabricated. Two spots labelled as spot 1 (without graphene covering) and spot 2 (with graphene covering) were selected to compare the optical behaviors of ZnO with/without graphene. Figure 3(d) exhibits the PL spectra from spot 1 and spot 2 under the same excitation condition. It can be seen that the intensity of the emission from spot 2 is about four times strong as that from spot 1, meanwhile, the FWHM is remarkably reduced after graphene being covered on the microbelt. This indicates that the graphene/ZnO hybrid microcavity is favorable for the lasing generation.

**Figure 4:**
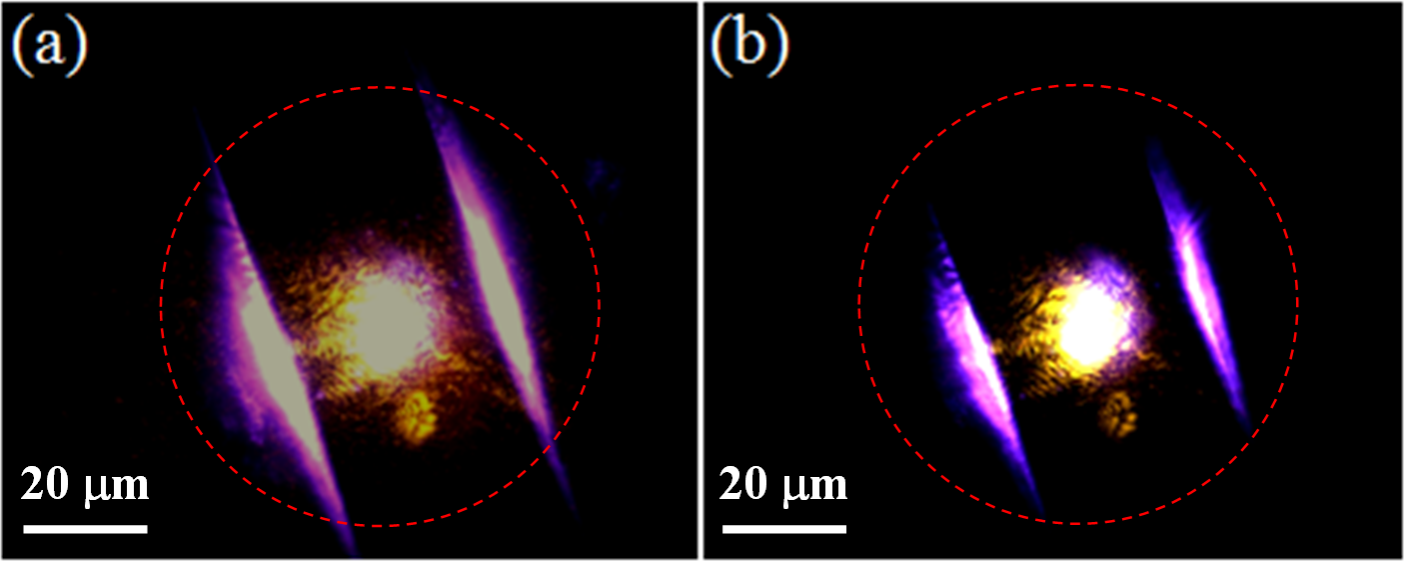
Dark field optical images of an individual ZnO microbelt (a) before and (b) after being covered with graphene under the same excitation of 325 nm UV laser.

Besides the enhanced PL intensity, an interesting phenomenon, i.e. the optical field confinement induced by graphene covering was also observed distinctly. As shown in Figure 4(a), when the bare ZnO microbelt was irradiated by 325 nm UV laser, it clearly displays a broad astigmatic emission spot, and the light emitted from the lateral sides of the microbelt propagates a proper distance along the length direction of the microbelt. While, as shown in Figure 4(b), when the same microbelt was covered with graphene and irradiated at the same excitation condition, the graphene/ZnO microbelt hybrid microcavity emits out much brighter blue-violet dazzling light, and the emission was almost confined in the close region of the excitation spot, showing obvious optical field confinement relative to that shown in Figure 4(a). This is a direct evidence for the optical field confinement induced by graphene SP.

**Figure 5:**
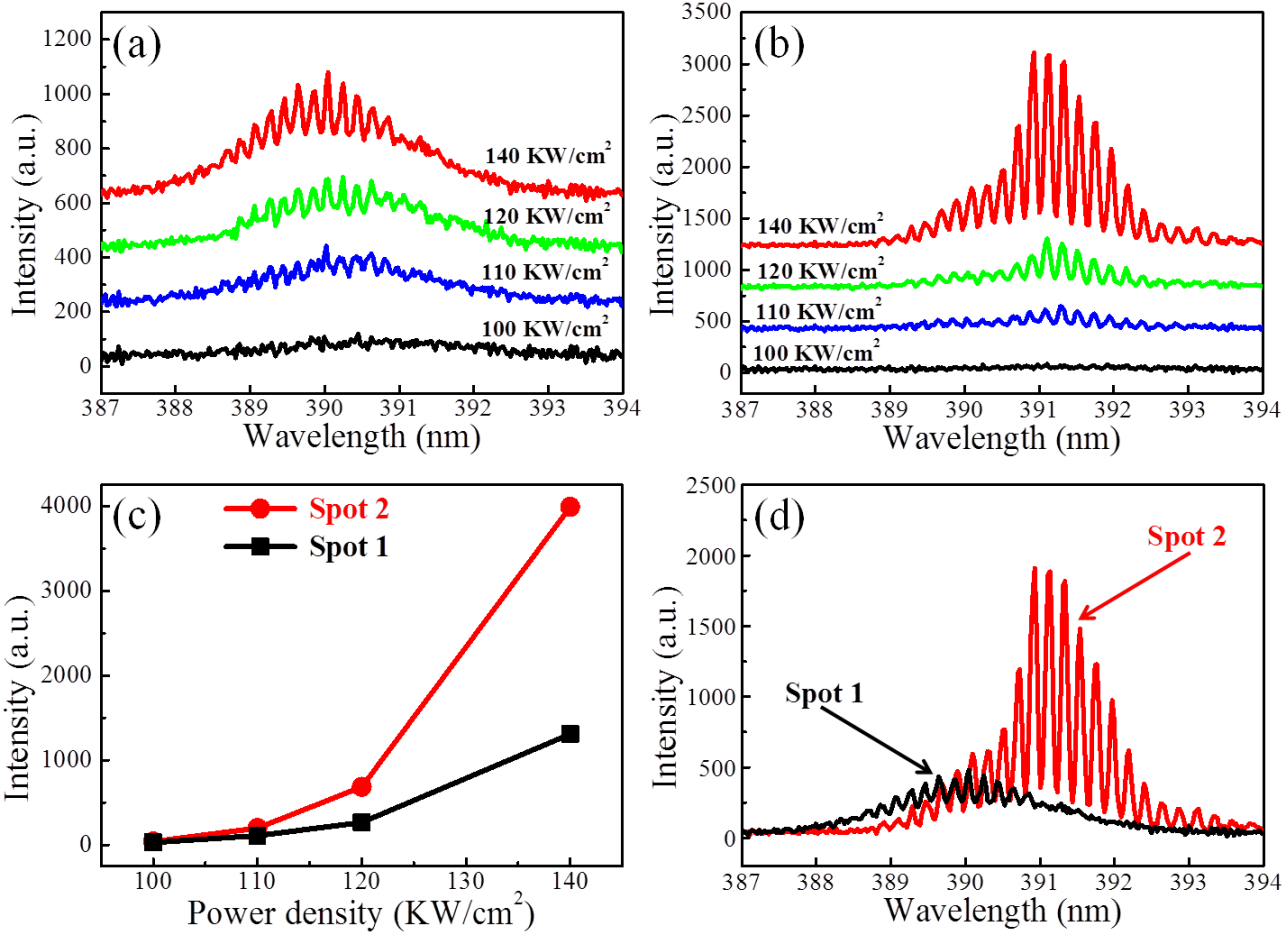
Excitation power density dependent PL spectra from (a) spot 1 and (b) spot 2. (c) Correlation between emission intensity and excitation power density for the bare and the graphene/ZnO microbelt hybrid microcavity. (d) Comparison of the lasing spectra from the two spots under the same excitation power density of 140 KW/cm^2^.

PL spectra from the bare and the hybrid microcavity were measured to investigate the impact of graphene SP on the lasing behaviors of the ZnO microbelt, as shown in Figures 5(a) and 5(b). Figure 5(a) shows the PL spectra from spot 1 under different excitation power densities. At a low excitation power density of 100 KW/cm^2^, the PL spectrum presents a broad spontaneous emission, which comes from the near band-edge (NBE) emission of ZnO^39^. Then the PL intensity increases slowly with increasing of the excitation power density. While, as the excitation power density reaches to 120 KW/cm^2^, some distinct and sharp peaks emerged in the spectrum, indicating the lasing generation from the bare ZnO microbelt. So we can deduce that the threshold for the bare microbelt is about 120 KW/cm^2^. The sharp peaks demonstrated distinctly the mode structure of the stimulated emission, while the broad and strong background indicates the optical loss and poor lasing quality, consistent with the simulation results demonstrated in Figures 2(a) and 2(b) and the experimental observation in Figure 4(a). For a typical lasing peak at wavelength of 390.2 nm, the mode spacing of the two adjacent lasing peaks is about 0.2 nm. According to the equation of mode spacing for F-P cavity Δ*λ* = (*λ*^2^/[2L(n−*λdn/dλ*)]^40^, where L is the cavity length, n = 2.47 is the refractive index of ZnO and *dn/dλ* = −0.026 nm^−1^ at *λ* = 390.2 nm denotes the dispersion relation for the refractive index, Δ*λ* = 0.2 nm as indicated by the PL spectrum, the calculated cavity length L = 30 μm, which agrees well with the experimental value measured by SEM. This indicates that the observed stimulated emission belong to the F-P lasing mechanism, which is formed in the cavity at the width direction of the cross-section of the microbelt. Actually, this F-P lasing mechanism also can be confirmed througth the dark field optical images of the bare ZnO microbelt and the hybrid microcavity. As shown in Figures 4(a) and 4(b), the microbelt emits bright blue-violet light from the lateral sides of the microbelt, this is a direct evidence for the F-P lasing resonant in the width direction of the cross-section of the microbelt, agreeing well with the calculation result discussed above and the simulation shown in Figure 2(a).

As the microbelt was covered with graphene, the PL spectra from spot 2 changed dramatically, as shown in Figure 5(b). With increasing of the excitation power densities, the PL spectra show similar evolution process from spontaneous emission to stimulated one, but the lasing peaks from spot 2 are much more pronounced than those from spot 1. For spot 2, when the excitation power density increased to 110 KW/cm^2^, distinct lasing peaks have appeared in the spectrum, indicating a lower threshold of spot 2 than that of spot 1. This can be attributed to the graphene SP's confinement of the optical field and its coupling with ZnO interband emission, as has been reported by our previous report^41^, where the WGM lasing enhancement and the obvious optical field confinement in a graphene/ZnO microrod hybrid microcavity have been systematically investigated through μ-PL mapping and the stable and transient PL experiments. In this present work, after graphene was covered on the microbelt, the crossover region of the excited evanescent wave of graphene SP and ZnO optical field could provide a platform to achieve the coupling between the graphene SP modes and conventional F-P resonant modes, so the total energy distribution and the oscillation process in the microcavity were modulated and the gain of the microcavity was improved, resulting in the lowered threshold of spot 2 relative to that of spot 1.

Figure 5(c) shows the lasing emission intensity as function of the excitation power density for the bare ZnO microbelt and the graphene/ZnO hybrid microcavity. Here, the kinks can be observed clearly for the threshold estimation. For the bare microbelt (spot 1 denoted), the PL intensity increases slowly with increasing of the excitation power density. When the excitation power density reaches to 120 KW/cm^2^, the slope increased sharply and some distinct and sharp peaks appeared in the spectrum as shown in Figure 5(a), indicating the occurrence of lasing from the bare ZnO microbelt. So we can deduce that the threshold for the bare microbelt is about 120 KW/cm^2^. The slow increase of the lasing intensity with increase of the excitation power density was caused by the optical loss of the bare microcavity, as shown in Figure 2(a), where a proper of light leaks out of the cavity at the ZnO/air interface to free space due to the poor confinement of the bare cavity. While for the graphene/ZnO hybrid microcavity (spot 2 denoted), the emission intensity increases rapidly as the excitation power density increased to 110 KW/cm^2^, the mode structure is clearer and the intensity of the emission is much higher than that of the bare microcavity at the same excitation condition. From this we can conclude that the threshold of the hybrid microcavity is 110 KW/cm^2^, which is lower than that of the bare one. It should be noted that the lasing emission intensity of the hybrid microcavtiy increase more quickly than that of the bare one, indicating the more efficient output of the microcavity due to the optical field confinement induced by graphene SP and its effective coupling with ZnO.

A clearer comparison of the lasing behaviors of the two spots under the same excitation of 140 KW/cm^2^ is shown in Figure 5(d). One can see clearly that the lasing spectrum from spot 2 obviously reveals sharper peaks and stronger lasing intensity than that from spot 1. This remarkably enhanced lasing intensity comes from the coupling between the graphene SP modes and the ZnO F-P resonant modes. It should be noted that a redshift of the lasing spectra profile has been observed as the ZnO microbelt was covered with graphene. This can be attributed to the band gap renormalization of ZnO^42^. In general, electron-hole plasma (EHP) lasing would be realized when the excitation intensity yielded a carrier density higher than Mott transition density^43^. In this present work, after the ZnO microbelt was covered with graphene, the exciton density can reach easily to the critical point for Mott transition due to the optical field confinement induced by graphene SP, so the EHP emission arises and the system energy decreases due to the band gap renormalization, resulting in the emission band redshifts to the longer wavelength.

In summary, a hybrid plasmonic F-P microcavity was constructed and fabricated by covering monolayer graphene on the ZnO microbelt, and unambiguously improved F-P lasing performance was realized, including the lowered lasing threshold, the improved lasing quality and the significantly enhanced lasing intensity. Theoretical simulation indicated that the graphene SP wave was excited along the interface of graphene/ZnO and the crossover region provide a platform to realize the coupling interaction between graphene SP modes and the ZnO F-P modes. On the other hand, the coupling interaction can play a modulation of the oscillation and energy storage of the ZnO F-P microcavity. The results presented here would be valuable for both understanding the interaction between graphene and semiconductor under the optical excitation in physics and motivating new ideas for designing of graphene-based optic and photoelectronic devices in technology.

## Methods

ZnO microbelts were synthesized by a VPT method^44^. In detail, a mixture of high purity ZnO and graphite powders (1:1 in mass ratio) was placed into a small quartz boat as source materials. A cleaned Si substrate was sputtered with 50 nm thick ZnO film by a radio frequency magnetron sputtering system and covered on the boat. The boat was then placed in the center of a quartz tube and the whole assembly was put into a horizontal tube furnace, which was heated to 1050°C. After growing for 50 min, the furnace was naturally cooled to room temperature. Then the microbelts can be found on the Si substrate and the inner surface of the quartz boat. It should be noted that a constant flow of high purity argon (100 sccm) was introduced into the tube furnace as protecting gas during the synthesis process. High quality monolayer graphene used in this experiment was synthesized by a chemical vapor deposition (CVD) method^45^. The hybrid microcavity was fabricated by transferring a piece of monolayer graphene on the ZnO microbelt with a standard method^46^. The morphology and structure of the samples were characterized by field emission scanning electron microscopy (FESEM, Carl Zeiss Ultra Plus) and optical microscopy (OLYMPUS BX53F). For optical measurements, the samples were excited by a focused 325 nm laser through a μ-PL system, and the incident lasing was focused to a spot size of ~15 μm. The spectra were collected by an optical multichannel analyzer (Princeton, Acton SP2500i). All measurements were performed at room temperature. Theoretical simulation was carried out by using a FDTD method.

## Author Contributions

C.X. Xu conceived and designed the experiments. J.T. Li and Y. Lin carried out the optically pumped experiments. Y.Y. Wang and J.F. Lu optimized the growth conditions for the ZnO microbelts and the CVD-grown graphene. M.M. Jiang carried out the simulations and theoretical analysis. Z.L. Shi gave scientific advice. J.T. Li and C.X. Xu wrote the paper and all authors commented on the manuscript at all stages.
